# Predicting a kidney transplant patient’s pre-transplant functional status based on information from waitlist registration

**DOI:** 10.1038/s41598-023-33117-y

**Published:** 2023-04-15

**Authors:** Ethan Mark, David Goldsman, Brian Gurbaxani, Pinar Keskinocak, Joel Sokol

**Affiliations:** grid.213917.f 0000 0001 2097 4943 H. Milton Stewart School of Industrial and Systems Engineering, Georgia Institute of Technology, Atlanta, GA USA

**Keywords:** Machine learning, Mathematics and computing, Kidney

## Abstract

With over 100,000 patients on the kidney transplant waitlist in 2019, it is important to understand if and how the functional status of a patient may change while on the waitlist. Recorded both at registration and just prior to transplantation, the Karnofsky Performance Score measures a patient’s functional status and takes on values ranging from 0 to 100 in increments of 10. Using machine learning techniques, we built a gradient boosting regression model to predict a patient’s pre-transplant functional status based on information known at the time of waitlist registration. The model’s predictions result in an average root mean squared error of 12.99 based on 5 rolling origin cross validations and 12.94 in a separate out-of-time test. In comparison, predicting that the pre-transplant functional status remains the same as the status at registration, results in average root mean squared errors of 14.50 and 14.11 respectively. The analysis is based on 118,401 transplant records from 2007 to 2019. To the best of our knowledge, there has been no previously published research on building a model to predict kidney pre-transplant functional status. We also find that functional status at registration and total serum albumin, have the most impact in predicting the pre-transplant functional status.

## Introduction

In 2019, there were over 100,000 kidney transplant patients aged 18 and over listed at the end of the year^1^. With 30% of patients aged 18 and over waiting 3 or more years to receive a decreased donor transplant in 2019, it is important to understand how the functional status of a patient changes during the potentially long time period between waitlist registration and transplantation. A patient’s pre-transplant functional status is an important predictor of post-transplant survival for kidneys^2–4^ and other organs^5,6^. Functional status has also been shown to predict the likelihood of receiving a kidney^7^ and survival in chronic kidney disease^8^. Furthermore, understanding potential changes in functional status while on the waitlist is also important to deciding whether to accept a deceased donor organ offer or remain on the waitlist for a potentially more optimal organ offer. While there has been strong previous research^9–13^ predicting kidney waitlist or transplant survival times (including likelihood of death on the waitlist and probability of never receiving a transplant), to the best of our knowledge, there is no previously published research on building a model to predict kidney pre-transplant functional status.

Using machine learning, we built a model that predicts the kidney pre-transplant functional status, given information known about the patient at waitlist registration. We also identified important predictive variables and provided a comparison of the predictive performance for different models and methods.

## Methods

### Data and data preparation

This study used data from the Scientific Registry of Transplant Recipients (SRTR)^14–16^. The SRTR data system includes data on all donor, wait-listed candidates, and transplant recipients in the US, submitted by the members of the Organ Procurement and Transplantation Network (OPTN). The Health Resources and Services Administration (HRSA), U.S Department of Health and Human Services provides oversight to the activities of the OPTN and SRTR contractors. The data consists of transplant records since October 1, 1987 until March 2, 2020. Information about requesting the data can be found at https://www.srtr.org/requesting-srtr-data/data-requests/. This study was granted exemption status by the Georgia Tech Institutional Review Board.

To prepare the dataset, we combined three Standard Analysis Files (SAF) provided by SRTR: cand_kipa.sas7bdat, which mostly contains information about transplant candidates when they are waiting to receive a kidney transplant; tx_ki.sas7bdat, which contained the transplant recipients’ pre-transplant functional status; and txf_ki.sas7bdat which contained transplant follow up information. We used all records where a patient was originally waitlisted for a kidney and received a deceased donor kidney transplant. We removed observations where the recipient’s age at listing was under 18 as well as observations with a missing pre-transplant functional status.

**Figure 1:**
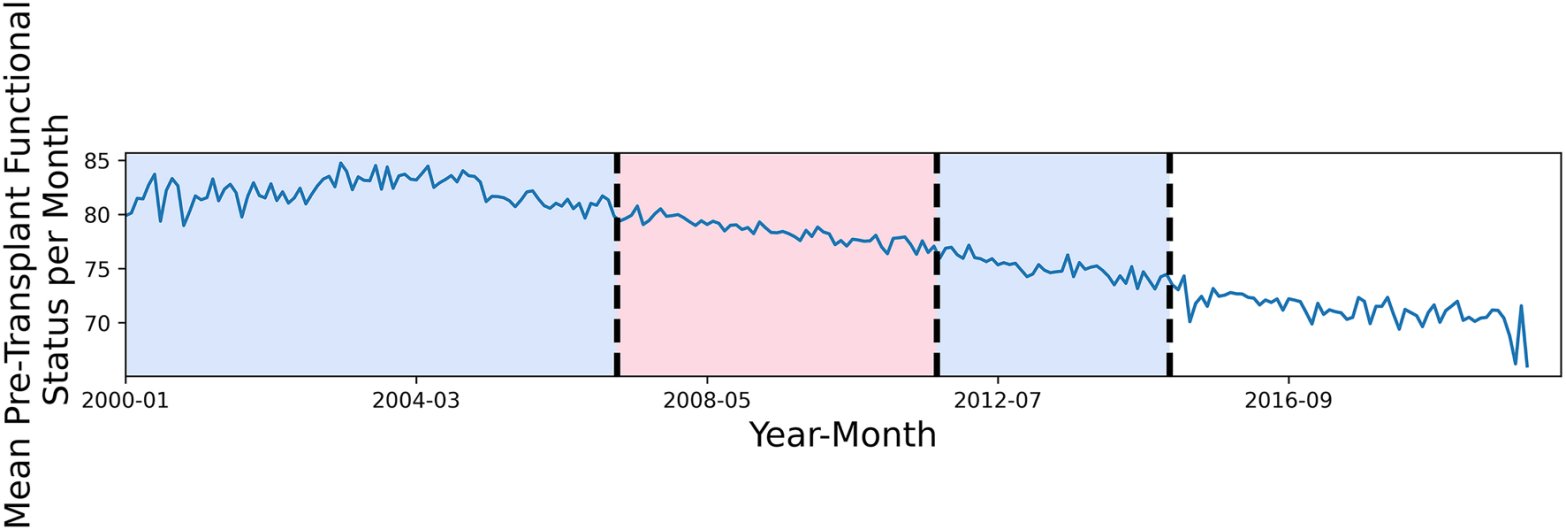
Average pre-transplant functional status per month, based on the month the patient registered on the waitlist, along with the points selected by the PELT change point detection algorithm. The data used for this plot consists of all observations where a patient was originally waitlisted for a kidney and received a deceased donor kidney transplant, where the recipient’s age at listing was over 18, and had a non-missing pre-transplant functional status.

In order to use the most up-to-date transplant records without discarding too many observations, we decided to select a cutoff date for being listed on the waitlist and filter out any observations where a patient was listed before this date. To determine the cutoff date, we used the change point detection algorithm, Pruned Exact Linear Time (PELT)^17^ with an L_2_ cost function and a penalty parameter of 100, to find the points in time of the most substantial changes in the data. We first calculated the average pre-transplant functional status per month for different patient waitlist registration dates and used the PELT algorithm on the monthly pre-transplant functional status, for all patients who were registered on the waitlist since January 1, 2000. The algorithm detected dates of February 2007, September 2011 and January 2015 (see Fig. 1). Using the earliest date detected, we selected 2007 as our cutoff date and filtered out patients that registered on the waitlist prior to 2007. We further used the two-sample Kolmogorov–Smirnov test^18^ to test the hypothesis that the distribution of pre-transplant functional status values for waitlist registration dates prior to 2007 was different than the distribution after 2007. We obtained a P-value of less than 0.001, suggesting that the distribution is different. Other possible cutoff dates we considered were December 2014, when United Network for Organ Sharing (UNOS) made changes to the kidney allocation system^19^. However we decided to use 2007 so that we did not remove too many observations and preserve useful information we would obtain from these transplants. Further, we did not use observations when a patient registered on the waitlist after 2019 because in order to include them, they would have needed to obtain a transplant prior to March 2, 2020. Since the average waiting times are much greater, including these observations would add sample bias since we would only be including transplants after 2019 that have had relatively short waiting periods. To have a more unbiased sample of 2019 transplants, we would want more years of data available. We selected 2019 as a cut-off date because we believed it balanced including the latest transplant data while preventing the use of biased transplant data.

Table 1 summarizes the inclusion and exclusion criteria we used to select the data. Unless otherwise stated, all analysis, figures and tables use the data based on this inclusion/exclusion criteria.

**Table 1:** Inclusion and exclusion criteria used to select the data.

Population size	Filter
971,974	Full dataset
470,867	Registered on the waitlist for a kidney transplant and received a kidney transplant
207,472	Waitlist registration date ≥ 2007-01-01
197,731	Waitlist registration date < 2019-01-01
190,862	Patient has a recorded pre-transplant functional status
124,839	Deceased donor transplant
118,401	Patient age at listing ≥ 18

We removed independent variables in the dataset if the information was not known at waitlist registration, based on the Adult Kidney Transplant Candidate Registration Worksheet^20^. We further removed variables with only one unique value, and categorical variables with more than 10 categories. Supplementary Table S1 contains a description of the variables considered for the predictive models and Supplementary Table S2 provides summary statistics of the variables.

To address missing data, for categorical variables, we created an additional category to denote if the value is missing. For numerical variables, prior to performing variable selection, we imputed variables using their median value. After variable selection, when building and cross-validating the predictive models, we used iterative imputation based on random forests^21^. We did not include pre-transplant functional status when imputing missing values, since it will not be known for new data that we want to predict. We further imputed each training and validation dataset separately to avoid data leakage. For categorical variables, we used one-hot-encoding (a new binary variable is created for each category of a categorical variable) where a reference category is dropped, when building the predictive models.

**Figure 2:**
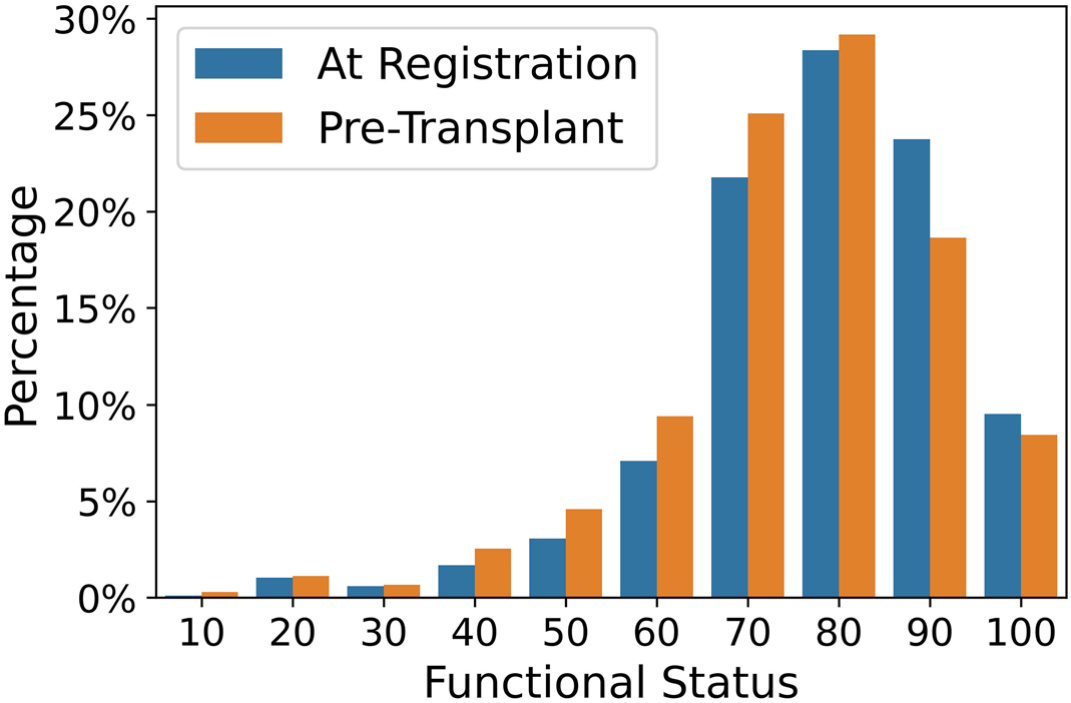
Distribution of the pre-transplant functional status and the status recorded at registration.

Our target variable in the data is ‘REC_FUNCTN_STAT’, which is the patient’s pre-transplant functional status from the Adult Kidney Transplant Recipient Registration Worksheet, recorded just prior to transplantation. The patient’s functional status is measured using the Karnofsky Performance Score (KPS)^22^ and takes on values ranging from 0 to 100 in increments of 10. The KPS has been shown to be a validated measure of functional status^23^ and a predictor of deceased donor kidney transplant outcomes^3^. The functional status is based on a patient’s ability to perform daily tasks and the amount of assistance they need. Example scores include “10%—Moribund, fatal processes progressing rapidly”, “100%—Normal, no complaints, no evidence of disease’, and “50%—Requires considerable assistance and frequent medical care”. Supplementary Table S3 lists the description of each value for pre-transplant functional status. Figure 2 illustrates the distribution of the pre-transplant functional status and the status at registration.

**Figure 3:**
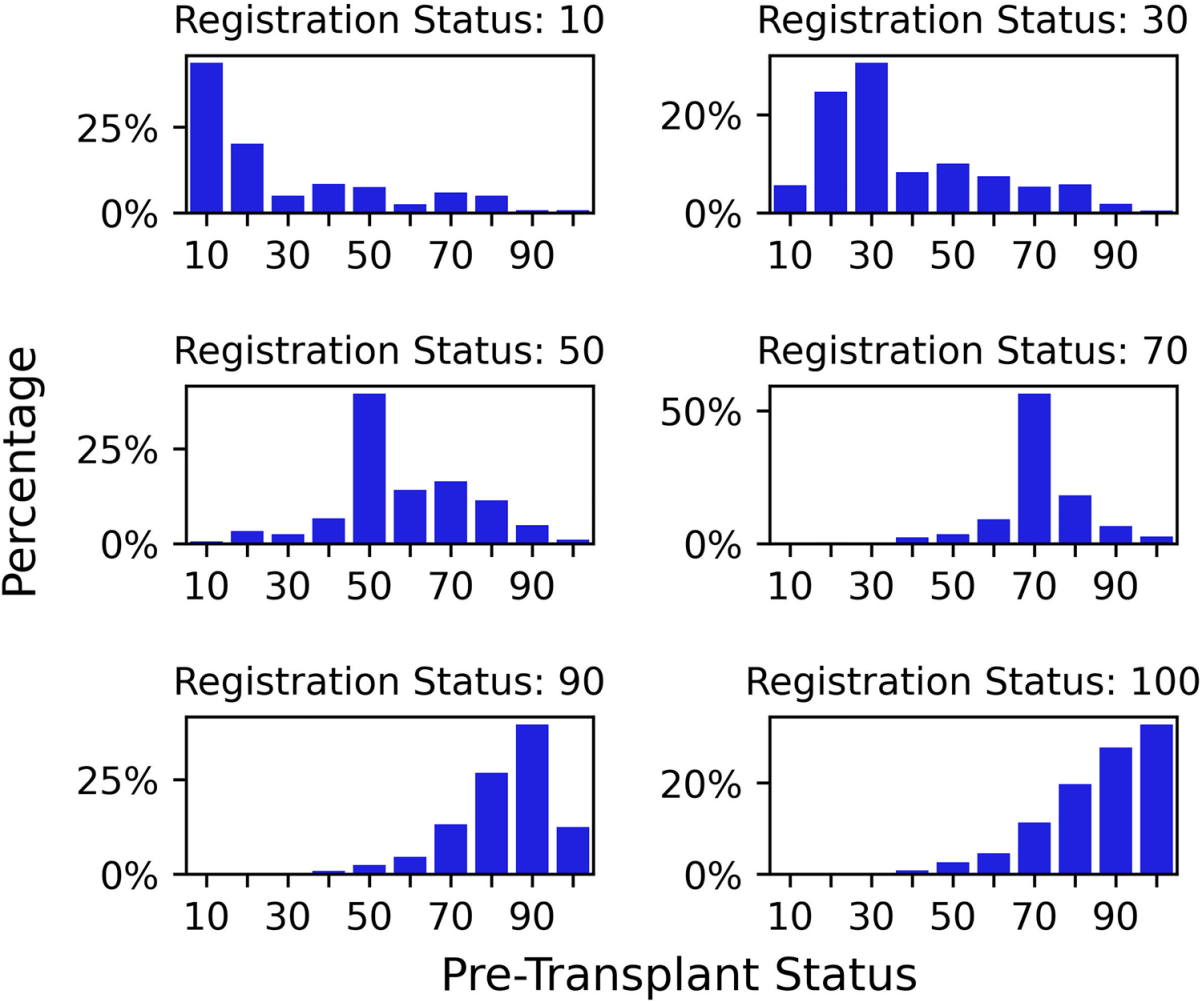
Distribution of the pre-transplant functional status by the status at registration.

Figure 3 shows the distribution of the pre-transplant functional status based on the status at registration. On average, patients with a functional status at registration at or above 80 have a lower pre-transplant status while patients with a functional status at registration below 80 have a higher pre-transplant status.

After we prepared the data, we split the data into a series of training and validations sets as well as an out-of-time set, based on the patient’s listing date on the waitlist. The training sets were used to train different predictive models and the validation sets were used to measure the performance of the models. Section “Building predictive models” provides more detail on the different training and validation sets we used.

### Feature engineering

In addition to using the original variables in the dataset, we performed feature engineering to create additional variables that may better represent the underlying relationship between the raw data and the target variable. Features created from the raw data include the quarter of the year when the patient was added to the waitlist, and the OPTN region based on the patient’s permanent state listed at registration^24^. In addition, we grouped the categories of the variable, Primary Diagnosis, from 75 categories to five different categories to reduce potential overfitting. In Supplementary Table S4 we show the original primary diagnosis values and their corresponding grouped categories. Supplementary Table S5 shows other relevant variable value groupings we performed to reduce having categories with few data points and potential overfitting.

We also checked variables for outliers and potential data entry errors. Several observations in the data may have been data entry errors, such as BMI values over 10,000. Hence, for the variables BMI and height, we set variable values larger or smaller than 6 standard deviations away from the mean as missing and used the imputation described in Section “Data and data preparation” to replace them.

### Selecting variables for the predictive models

We selected which variables to use for the predictive models using two different variable selection methods. The first method we used is permutation variable importance from Extreme Gradient Boosting^25^ which we denote as VIXGB. In this method, we first split the data into a training and a validation set. An Extreme Gradient Boosting (XGBoost) model was trained on the training data and its performance is recorded on the validation data. Then, for each variable, we permute its value to another random variable value in the validation set and recalculate its performance on the permuted validation data. For each variable, we repeat this process multiple times and take the average change in performance between the permuted validation data and the original validation data. The mean decrease in performance is the permutation importance. To help determine how many variables to use, we added two random variables following a normal distribution to the variable importance calculation. We then ranked each variable by its permutation importance calculation. Ten permutation for each variable were used and we used root mean squared error (RMSE) to measure the performance. The out-of-time data was left out of the permutation importance calculation to ensure no data leakage when testing the performance of our models. Of the non-out of time data, the first 80% of the observations was used for training and the last 20% was used for validation in the VIXGB calculation.

To investigate feature interactions, we computed Friedman and Popescu’s H statistics^26^ for all numerical pairs of variables of the top 15 variables chosen by VIXGB. The larger the H statistic, the stronger the evidence that there is an interaction between the two features. A gradient boosting^27,28^ model was used to calculate the H statistic and the out-of-time data was left out of the H statistic calculation.

After computing the variable importance metrics, we created five different sets of variables. These sets were: the top 5, the top 10, the top 15 and all variables ranked above the two random variables, from VIXGB, and the top 5 interactions chosen from the H statistics along with the top 5 variables chosen from VIXGB. We refer to our five sets of variables sets as (i) VIXGB Top 5, (ii) VIXGB Top 10 (iii) VIXGB Top 15, (iv) VIXGB Better than Random and (v) Interactions. These variable sets were used to cross-validate different model and variable set combinations.

### Building predictive models

To select our final model, we rigorously cross-validated different model and variable combinations. The predictive regression models we compared are: elastic net^29^, random forests^30^, Light Gradient Boosted Machines^31^ (LightGBM), Extreme Gradient Boosting (XGBoost)^25^, Generalized Additive Model (GAM)^32,33^ with an identity link function and a normal error distribution, and support vector regression^34^ with Nystrom approximation^35,36^.

In addition to approaching the problem from a regression framework, we also cross-validated a model from a classification approach since the ten different functional status values can also be modelled as 10 separate classes. For the classification approach we used an XGBoost classification model. Since the functional status values are ordered, it can also be modelled as a discrete ordered variable, hence we also built and tested an ordinal logistic regression model^37^.

For each of the eight models, we tested their performance using different sets of variables chosen by the variable selection methods in Section “Selecting variables for the predictive models”, as well as different hyper parameter combinations. After cross-validating each model and variable combination, we built a stacking model that combined the predictions of an elastic net, XGBoost, and LightGBM model. For the three sub models of the stacking model, we used the parameters that resulted in the lowest RMSE from the cross-validation results.

To compare our machine learning models to a benchmark model, we also tested the performance of two rule-based models. The first rule-based model predicts that the pre-transplant functional status will remain the same as the status at registration. Since 46.6% of the observations (without a missing pre-transplant or registration functional status) in the data have the same pre-transplant functional status as the status at registration, this simple benchmark model has a reasonable predictive power. The second rule-based model predicts that the functional status will shift from it’s value at registration by the average amount the functional status shifts from registration to pre-transplantation in the training data population. We denote these models ‘same as registration’ and ‘average shift’ in our results, and we will refer to the former as the benchmark model. In total, 251 different model, parameter and variable combinations were tested. For these two rule-based models, missing values of functional status at registration were imputed using the top 5 variables at from VIXGB.

Prior to building each predictive model we normalized the data for all models except the two rule-based models and support vector regression models (which used Nystrom approximation to transform the data). We used Z-score normalization, where the means and standard deviations were taken from the training data set. After building and making predictions with each model, the predictions were rounded to the nearest 10 to be on the same scale as the true functional status values, which are recorded in increments in 10.

**Figure 4:**
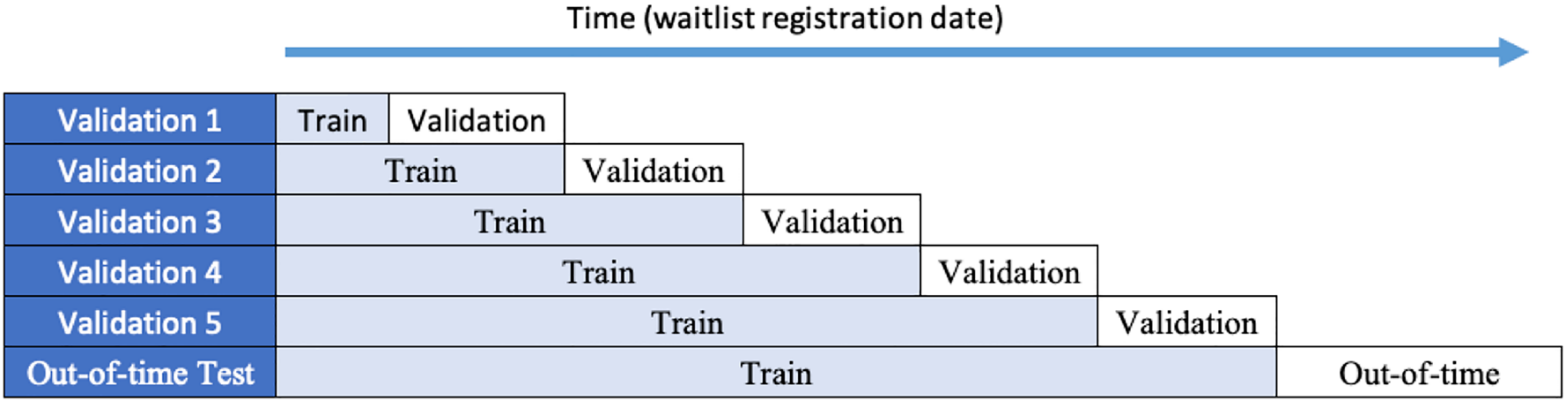
Cross-validation scheme using 5 ROCV tests and an out-of-time test.

To cross validate each of the predictive models, we used five ROCV train/validation test. In the first split, the train and validation sets are split into a roughly equal number of days, with the training data starting from the first date in the data set, and the validation set occurring after the latest date in the training data, to ensure that we are not using any information from future data to predict previous time periods. In the next cross validation split, the training and validations sets from the previous cross validation, become the new training data, and the new validation set begins immediately after the latest date in the new training set, and spans the same number of days as in the previous validation set. This process continues for five different cross validation sets, where the training dataset grows in each test and the origin of the validation set begins where the training data ends. After performing five ROCV tests on each predictive model, we tested our final model and the benchmark model again on an out of time data set to ensure consistent performance. The out-of-time data was selected to be the latest 20% days in the dataset. Table 2 shows the start and end dates of each train/validation split in addition to the out-of-time test. Note, that some days in the dataset do not have any observations so the first observation in a cross-validation split may occur a few days after the cross validation split start date. We also performed an additional test on the final and benchmark model by performing 20 ROCV tests on the full dataset to obtain a more in-depth model comparison. Figure 4 visualizes our cross-validation scheme.

**Table 2:** Train/validation splits for the 5 ROCV samples and the additional out-of-time test.

Cross validation split	Train start (inclusive)	Train end (inclusive)/validation start (exclusive)	Test end (inclusive)	Train size	Test size
1	2007-01-02	2008-07-11	2010-01-17	16,984	16,134
2	2007-01-02	2010-01-17	2011-07-26	33,118	16,153
3	2007-01-02	2011-07-26	2013-01-31	49,271	15,418
4	2007-01-02	2013-01-31	2014-08-09	64,689	15,710
5	2007-01-02	2014-08-09	2016-02-15	80,399	14,301
Out of time	2007-01-02	2016-02-15	2018-12-31	94,700	23,701

For the cross validations we used the following metrics to assess predictive accuracy: (i) RMSE: root mean squared error, (ii) σ RMSE: the standard deviation of RMSE for the cross-validation samples. (iii) A10: if the true value is X, the percent of predictions that are within (X-10 to X + 10 inclusive) and (iv) A20: if the true value is X, the percent of predictions that are within (X − 20 to X + 20 inclusive), Note that RMSE penalizes inaccurate predictions more severely compared to A10 or A20; for example, if the true value of an observation is 70, A10 penalizes a prediction of 50 the same as a prediction of 10. Nevertheless, A10 or A20 may be of interest to physicians or patients in practice. When performing ROCV, the average values of RMSE, A10 and A20 was computed on the different cross-validation tests. The analysis was completed using the programming language Python 3.8.2, and Supplementary Table S6 shows the functions and packages used in the major computations.

## Results

**Figure 5:**
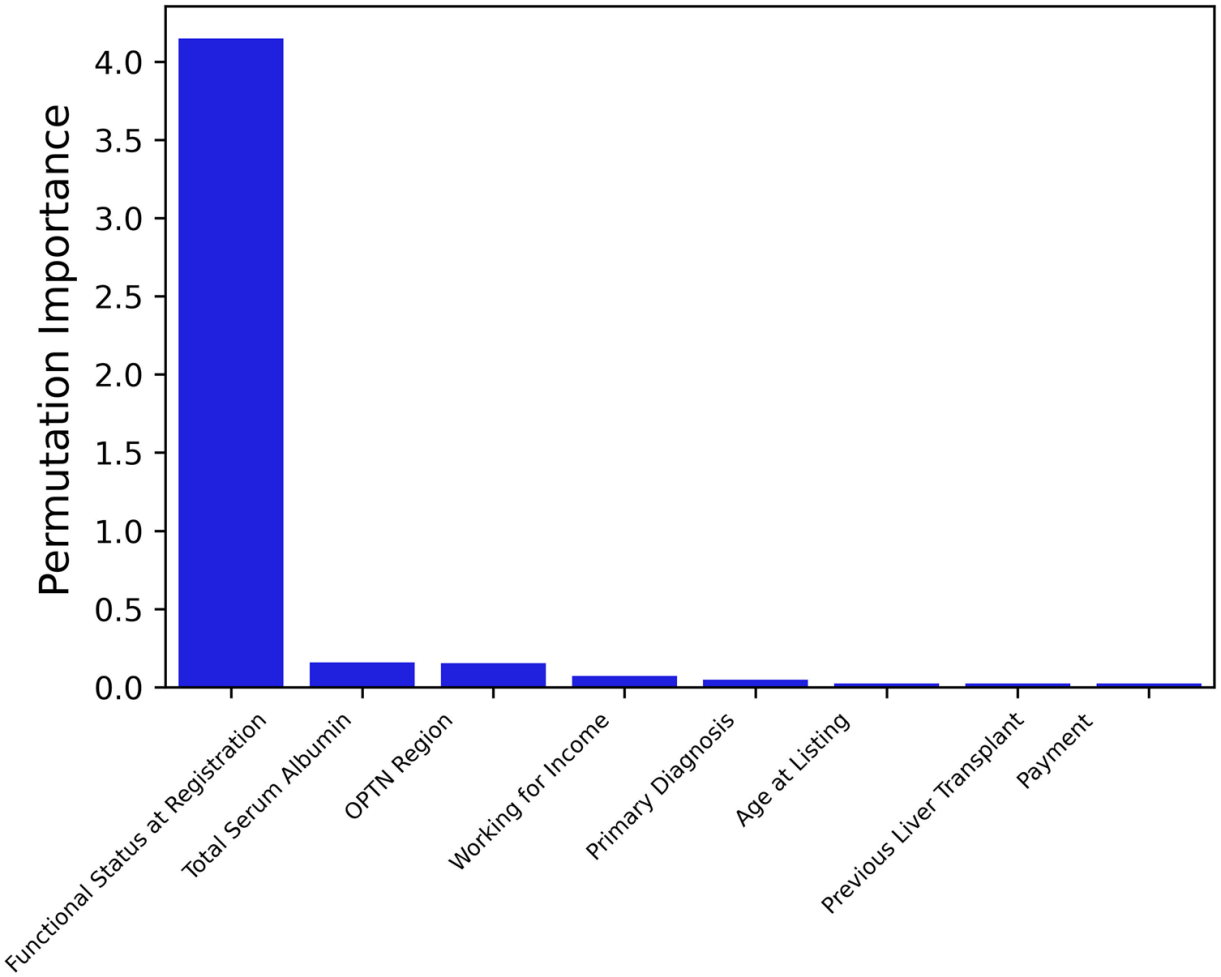
Top eight variables ranked by permutation importance using XGBoost.

Figure 5 shows the top 8 ranked variables by VIXGB and their respective importance measures. Functional status at registration was ranked as the most-important predictive variable by VIXGB. The next two most-important variables by permutation importance were total serum albumin, and OPTN Region. Table 3 shows the variables selected by each variable selection method.

**Table 3:** Variables selected by each of the variable selection methods. In the table, X,Y indicates an interaction between the variables X and Y.

VIXGB Top 5	VIXGB Top 10	VIXGB Top 15	VIXGB better than random variables	Interactions
CAN_DGN	AGE_AT_LISTING	AGE_AT_LISTING	AGE_AT_LISTING	CAN_BMI, CAN_WGT_KG
CAN_FUNCTN_STAT	CAN_ABO	CAN_ABO	CAN_ABO	CAN_DGN
CAN_TOT_ALBUMIN	CAN_BMI	CAN_BMI	CAN_BMI	CAN_FUNCTN_STAT
CAN_WORK_INCOME	CAN_DGN	CAN_DGN	CAN_DGN	CAN_HGT_CM, AGE_AT_LISTING
OPTN_REGION	CAN_FUNCTN_STAT	CAN_EDUCATION	CAN_DIAB_TY	CAN_HGT_CM, CAN_WGT_KG
	CAN_PREV_LI	CAN_ETHNICITY_SRTR	CAN_EDUCATION	CAN_TOT_ALBUMIN
	CAN_PRIMARY_PAY	CAN_FUNCTN_STAT	CAN_ETHNICITY_SRTR	CAN_TOT_ALBUMIN, AGE_AT_LISTING
	CAN_TOT_ALBUMIN	CAN_HGT_CM	CAN_EXHAUST_PERIT_ACCESS	CAN_WGT_KG, AGE_AT_LISTING
	CAN_WORK_INCOME	CAN_PERIPH_VASC	CAN_FUNCTN_STAT	CAN_WORK_INCOME
	OPTN_REGION	CAN_PREV_LI	CAN_GENDER	OPTN_REGION
		CAN_PRIMARY_PAY	CAN_HGT_CM	
		CAN_TOT_ALBUMIN	CAN_MALIG	
		CAN_WGT_KG	CAN_MALIG_TY_BREAST	
		CAN_WORK_INCOME	CAN_MALIG_TY_CNS_TUMOR	
		OPTN_REGION	CAN_MALIG_TY_GENITOURINARY	
			CAN_MALIG_TY_LEUK_LYMPH	
			CAN_MALIG_TY_LIVER	
			CAN_MALIG_TY_LU	
			CAN_MALIG_TY_SKIN_MEL	
			CAN_MALIG_TY_THROAT	
			CAN_MALIG_TY_THYROID	
			CAN_MALIG_TY_UNK	
			CAN_PERIPH_VASC	
			CAN_PREV_HL	
			CAN_PREV_HR	
			CAN_PREV_IN	
			CAN_PREV_KI	
			CAN_PREV_KI_TX_FUNCTN	
			CAN_PREV_KP	
			CAN_PREV_LI	
			CAN_PREV_LU	
			CAN_PREV_PA	
			CAN_PREV_TX	
			CAN_PRIMARY_PAY	
			CAN_TOT_ALBUMIN	
			CAN_WGT_KG	
			CAN_WORK_INCOME	
			LISTING_QUARTER	
			OPTN_REGION	

The RMSE, standard deviation of RMSE, and accuracy of classification within one and two increments for the top 10 models along with the two rule-based models are shown in Table 4. The table shows the average and standard deviation of the metrics across the 5 different cross-validation tests. The XGBoost model with the top 15 variables from VIXGB resulted in the lowest average RMSE based on the five cross-validation samples. This model, with an average RMSE of 12.99, performed much better than the benchmark model with an average RMSE of 14.50 based on the 5 ROCV tests. It also obtained an average accuracy of classification within one increment of 80.19% and accuracy within two increments of 94.24% (compared to 77.65% and 91.05%, respectively, for the benchmark model). The model further performed better in the out-of-time test with an RMSE of 12.94 vs. 14.11.

**Table 4:** Performance of the top 10 predictive models along with the two rule-based models, from five ROCV tests.

RMSE validation	σ RMSE validation	Accuracy within one increment validation	Accuracy within two increments validation	RMSE train	Model parameters
12.99	0.682	80.19%	94.24%	11.81	Model:XGBoost, features:VIXGB Top 15, tree_method:auto, objective:reg:squarederror, booster:gbtree, learning_rate:0.01, gamma:0, max_depth:8, colsample_bytree:0.8, colsample_bynode:0.3, subsample:1, min_child_weight:1.0, n_estimators:500
13.0	0.679	80.17%	94.26%	11.87	Model:XGBoost, features:VIXGB Top 15, tree_method:auto, objective:reg:squarederror, booster:gbtree, learning_rate:0.01, gamma:0, max_depth:8, colsample_bytree:0.8, colsample_bynode:0.3, subsample:1, min_child_weight:3.0, n_estimators:500
13.02	0.709	80.51%	94.18%	12.18	Model:LightGBM, features:VIXGB Better than Random Variables, objective:regression, boosting_type:gbdt, learning_rate:0.1, num_leaves:32, min_child_samples:20, max_depth:-1, colsample_bytree:0.9, subsample:1, n_estimators:50
13.03	0.661	80.13%	94.26%	12.03	Model:XGBoost, features:VIXGB Top 10, tree_method:auto, objective:reg:squarederror, booster:gbtree, learning_rate:0.01, gamma:3, max_depth:8, colsample_bytree:0.8, colsample_bynode:0.3, subsample:1, min_child_weight:0.4, n_estimators:500
13.03	0.659	80.3%	94.25%	11.61	Model:XGBoost, features:VIXGB Top 10, tree_method:auto, objective:reg:squarederror, booster:gbtree, learning_rate:0.01, gamma:0, max_depth:10, colsample_bytree:0.8, colsample_bynode:0.3, subsample:1, min_child_weight:0.4, n_estimators:500
13.03	0.701	80.47%	94.16%	12.21	Model:LightGBM, features:VIXGB Top 15, objective:regression, boosting_type:gbdt, learning_rate:0.01, num_leaves:32, min_child_samples:20, max_depth:-1, colsample_bytree:0.9, subsample:1, n_estimators:500
13.05	0.689	80.42%	94.18%	12.31	Model:LightGBM, features:VIXGB Top 10, objective:regression, boosting_type:gbdt, learning_rate:0.01, num_leaves:32, min_child_samples:20, max_depth:-1, colsample_bytree:0.9, subsample:1, n_estimators:500
13.06	0.642	80.12%	94.18%	11.37	Model:XGBoost, features:VIXGB Top 15, tree_method:auto, objective:reg:squarederror, booster:gbtree, learning_rate:0.1, gamma:0, max_depth:10, colsample_bytree:0.8, colsample_bynode:0.3, subsample:1, min_child_weight:3.0, n_estimators:50
13.06	0.712	80.13%	94.19%	12.38	Model:LightGBM, features:VIXGB Top 15, objective:regression, boosting_type:gbdt, learning_rate:0.01, num_leaves:32, min_child_samples:20, max_depth:-1, colsample_bytree:0.9, subsample:1, n_estimators:300
13.06	0.693	80.5%	94.1%	11.95	Model:LightGBM, features:VIXGB Top 15, objective:regression, boosting_type:gbdt, learning_rate:0.1, num_leaves:32, min_child_samples:20, max_depth:-1, colsample_bytree:0.9, subsample:1, n_estimators:100
14.5	0.784	77.65%	91.05%	14.22	Model:same as registration
14.51	0.767	77.47%	90.99%	14.19	Model:average shift

The metrics shown for each model are the average and standard deviation from the five different cross-validation tests.

Table 5 shows a side-by-side comparison of the performance tests of the final model and the benchmark model for each cross-validation test. In our additional model tests between the final model and the benchmark model (see Supplementary Table S7), a two-sided paired t-test comparing the RMSE for each of the 20 cross validation samples yielded a p-value of 3.72*10^−18^, providing strong evidence that there is a significant difference between the final model and the benchmark model. Supplementary Table S8 shows the performance of the best performing parameters from each model type.

**Table 5:** Comparison of the final model to the benchmark model for each cross-validation test on the validation data and on the out-of-time data test.

Validation	Final model RMSE	Final model accuracy within one increment	Final model accuracy within two increments	Benchmark RMSE	Benchmark accuracy within one increment	Benchmark accuracy within two increments
1	12.3	81.81%	95.53%	13.87	78.99%	91.81%
2	12.34	81.84%	95.6%	13.64	79.41%	92.84%
3	12.96	80.24%	94.17%	14.38	77.52%	91.55%
4	13.57	78.72%	93.17%	15.19	76.27%	89.72%
5	13.79	78.35%	92.73%	15.42	76.04%	89.34%
Out of time	12.94	81.49%	93.96%	14.11	79.88%	91.38%

In addition to recording the performance of the model on the entire data population, we recorded the model performance for different segments of the population in Supplementary Table S9. In the segment analysis, the model was trained on the non out-of-time data and tested on the out-of-time data. For each categorical variable, the performance was tested on each of the different variable categories and for numerical variables, the performance was tested on the first third, second third, on last third percentiles of values. The table shows that the model performs better than the benchmark model for all segments of the population except for observations with an OPTN region category of ‘other’ and observations in OPTN region 9. However, these represent relatively small segments of the population, with only 52 observations with an OPTN region category of ‘other’ and 1740 in region 9 from the out-of-time data. Further, the difference in RMSE is only 10.45 vs. 10.13 for the final model and the benchmark model amongst observations in region 9. The segment analysis can provide insight for researchers looking to make further improvements to the model in the future.

**Figure 6:**
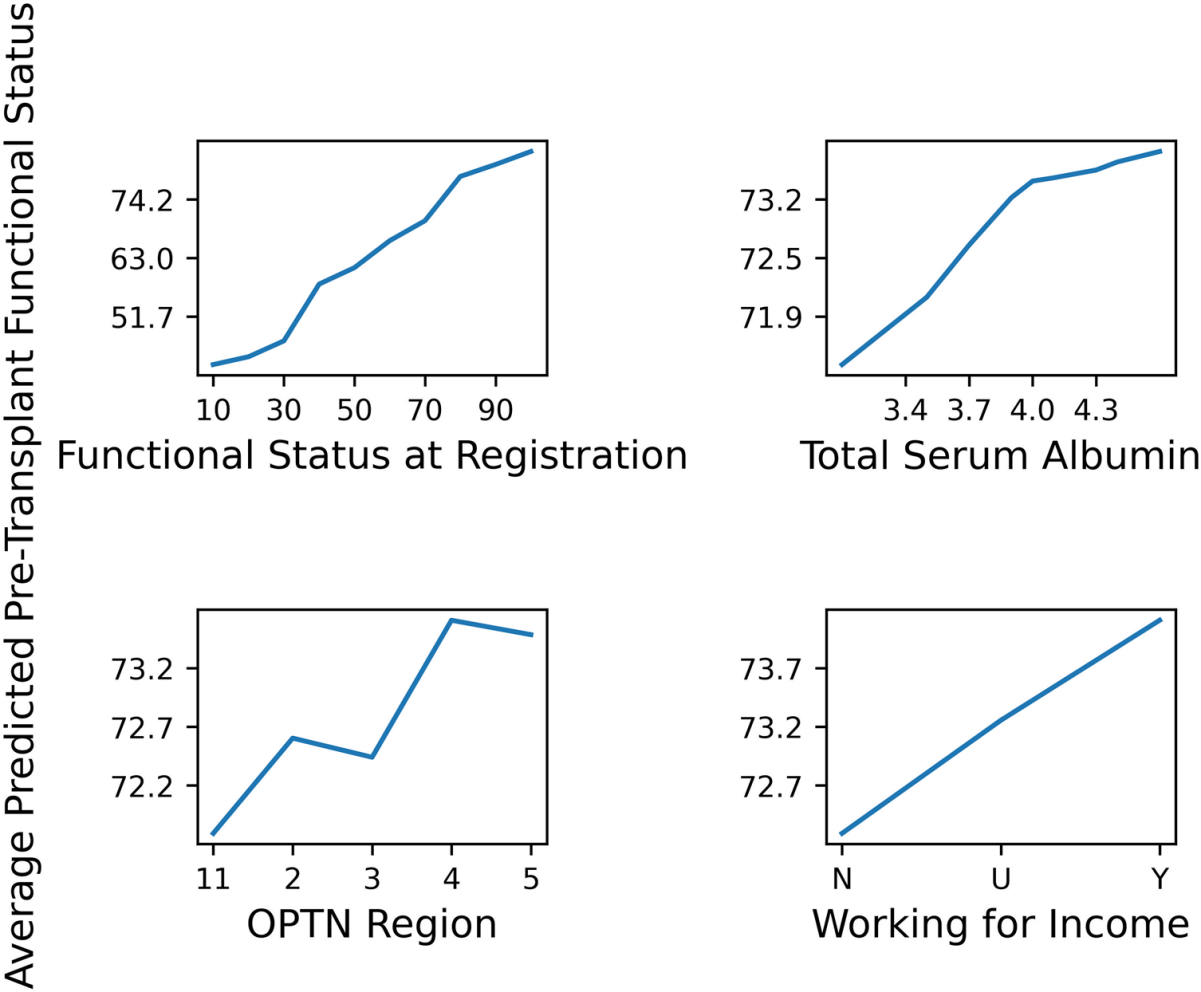
Partial Dependence Plots of the top four ranked variables by permutation importance. To produce these plots for one variable, we first trained the model on the non out-of-time data. In the out-of-time data, we then set the value of the variable to a particular value for all observations, while the rest of the variables were left unchanged and plotted the model’s average predictions for the modified out-of-time data. We repeated this process for different variable values.

To illustrate the behavior of the model, we produced partial dependence plots for the top four ranked variables by permutation importance in Fig. 6. The partial dependence plots show how the predicted value of our model changes when we change the value of one variable and hold the rest constant. The plots show that as we increase the functional status at registration, the model’s predictions for the pre-transplant functional status also increase, and that the association is very strong. We also find that there is a positive association between total serum albumin and the model’s predicted value. Likewise, being able to work for income is also associated with a higher pre-transplant functional status. The plots further show that different OPTN regions result in small differences in the predicted values.

## Discussion

We built models to predict the pre-transplant functional status, given information known at waitlist registration. The model can be used to predict how the functional status of a patient may change while on the waitlist, from registration to transplantation. The model performs significantly better, across multiple metrics, compared to a benchmark model which assumes that the functional status will remain the same between registration and transplantation.

**Figure 7:**
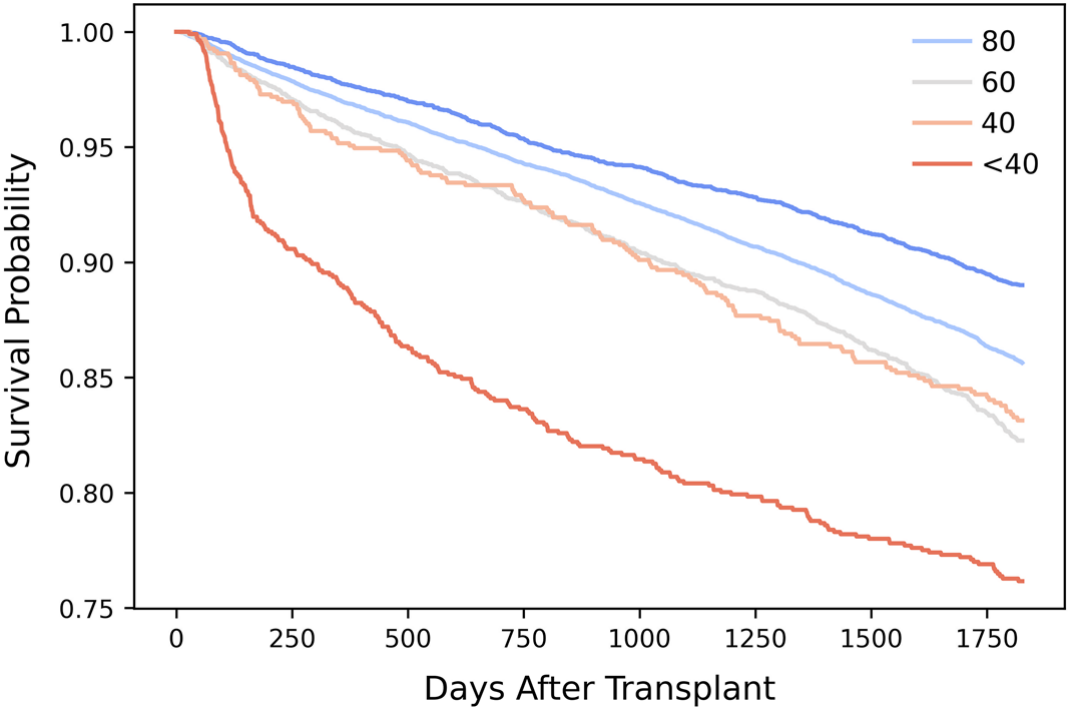
Post-transplant survival for different groups of patients based on their pre-transplant functional status using the Kaplan–Meier estimate. In the Kaplan–Meier estimate, the survival time was the time between the transplant date (REC_TX_DT) and the latest follow up date (TFL_PX_STAT_DT). An observation was considered censored if the patient status (TFL_PX_STAT) at the latest follow up date was living or retransplanted. Observations with a status that is ‘lost to follow up’ or ‘not seen’ were removed. The data used for this figure have the same inclusion/exclusion criteria as shown in Table 1 except that we removed observations with a transplant date after March 1, 2015 so that we have at least five years of data between the transplant date and latest patient follow-up date of March 2, 2020.

Predicting changes in functional status could be helpful especially when a patient is offered a deceased donor organ, while assessing the tradeoffs between accepting the offer and having a transplant, versus remaining on the waitlist for a potentially better organ offer that may arrive in the future. Pre-transplant functional status is an important predictor of kidney transplant survival (controlling for other variables)^38^, and hence, the decision to remain on the waitlist needs to consider the possibility of a change in functional status while on the waitlist. Figure 7 visualizes how higher pre-transplant functional status is associated with higher post-transplant survival using the Kaplan–Meier estimate^39^. Pre-transplant functional status predications can also be helpful in estimating potential healthcare costs and caregiving needs^40^.

Our model focuses on predicting the functional status of a patient just prior to transplantation; it does not predict the likelihood of a patient dying while on the waitlist, nor does it predict the likelihood (and timing) of a patient to receive a transplant in the future. In our dataset, 19% of patients aged 18 and older who were listed for the kidney waitlist between January 1 2007 to January 1 2019 were removed from the waitlist because they died or were too sick to receive a transplant. Models predicting the likelihood of transplantation^41^ or death^12^ before kidney transplantation, can be found in the literature, and be used in conjunction with the model proposed here, e.g., while evaluating decisions regarding organ offers.

A limitation of this analysis is that KPS has been reported to have some subjectivity in its recording^42^. A patient’s true health status may be slightly different than their reported status. On the positive side, the usefulness of KPS in predicting post-transplant survival and the likelihood of receiving a kidney has been established in previous literature^2–7,43^. Further, other interest in predicting the KPS is illustrated by Kok et al.^44^ where the authors predict functional status after transplantation for patients with acute-on-chronic liver failure. Hence, even if there is some subjectivity in the recording of KPS at registration, the high predictive accuracy of our model would still lead to useful information and insights for patients and physicians.

Another potential application of this study is to help determine which patients might benefit from prehabilitation. Candidates with low predicted pre-transplant functional status may benefit from prehabilitation to improve their functional status between waitlist registration and pre-transplantation. Examples of prehabilitation include aerobic or cardiovascular exercises, strength training exercises using resistance bands or weights, stretching, and trampoline exercises to promote coordination^45^. Additional prehabilitation can also include nutritional support or education related to physical activity^46^. Research has suggested that prehabilitation for patients on the kidney transplant waitlist is feasible and may improve post-transplant outcomes^45^. Future research can explore how the predicted pre-transplant functional status and SRTR data could be utilized to identity candidates with a high potential to benefit from prehabilitation, or to assess which prehabilitation activities can be most beneficial considering a candidate’s characteristics.

The proposed model utilizes data from SRTR. Additional data on a patient’s lifestyle (e.g. nutrition, physical activity) can be incorporated into a more complex model for future research.

## Supplementary Information

Supplementary Tables.

## Data Availability

This study used data from the Scientific Registry of Transplant Recipients (SRTR). The SRTR data system includes data on all donor, wait-listed candidates, and transplant recipients in the US, submitted by the members of the Organ Procurement and Transplantation Network (OPTN). The Health Resources and Services Administration (HRSA), U.S Department of Health and Human Services provides oversight to the activities of the OPTN and SRTR contractors. The data consists of transplant records since October 1, 1987 until March 2, 2020. Information about requesting the data can be found at https://www.srtr.org/requesting-srtr-data/data-requests/.
