# Supplementary material for: Predicting a kidney transplant patient’s pre-transplant functional status based on information from waitlist registration
Source: Sci Rep. 2023 Apr 15;13:6164. doi: 10.1038/s41598-023-33117-y (PMC10105757; doi:10.1038/s41598-023-33117-y)
Supplement: Supplementary file 1 — Supplementary Tables. [file 41598_2023_33117_MOESM1_ESM.docx]

**Supplementary information for predicting a kidney transplant patient’s pre-transplant functional status based on information from waitlist registration**

Ethan Mark^1^, David Goldsman^1^, Brian Gurbaxani^1^, Pinar Keskinocak^1*^, Joel Sokol^1^

^1^H. Milton Stewart School of Industrial and Systems Engineering, Georgia Institute of Technology, Atlanta, Georgia, U.S.A.

*Corresponding author: P. Keskinocak

| Variable | Description | Categories |
| --- | --- | --- |
| AGE_AT_LISTING | Candidate Age at Listing |  |
| CAN_ABO | Patient/s Blood Type | A, A1, A1B, A2, A2B, AB, B, O |
| CAN_BMI | BMI |  |
| CAN_CITIZENSHIP | Patient/s Citizenship | NAN, NON-RESIDENT ALIEN, YEAR ENTERED US, NON-US CITIZEN/NON-US RESIDENT, TRAVELED TO US FOR REASON OTHER THAN TRANSPLANT, NON-US CITIZEN/NON-US RESIDENT, TRAVELED TO US FOR TRANSPLANT, NON-US CITIZEN/US RESIDENT, RESIDENT ALIEN, US CITIZEN |
| CAN_DGN | Primary Diagnosis / KI Diagnosis for KP Candidate | GROUP_0, GROUP_1, GROUP_2, GROUP_3, GROUP_4 |
| CAN_DIAB_TY | Diabetes Type I/II | DIABETES STATUS UNKNOWN, NAN, NO, TYPE I, TYPE II, TYPE OTHER, TYPE UNKNOWN |
| CAN_EDUCATION | Patient/s Educational Status | ASSOCIATE/BACHELOR DEGREE, ATTENDED COLLEGE/TECHNICAL SCHOOL, GRADE SCHOOL (0-8), HIGH SCHOOL (9-12) OR GED, N/A (< 5 YRS OLD), NAN, NONE, POST-COLLEGE GRADUATE DEGREE, UNKNOWN |
| CAN_ETHNICITY_SRTR | SRTR Patient Ethnicity | LATINO, NON-LATINO OR UNKNOWN |
| CAN_EXHAUST_PERIT_ACCESS | Exhausted Peritoneal Access | N, U, Y |
| CAN_EXHAUST_VASC_ACCESS | Exhausted Vascular Access | N, U, Y |
| CAN_FUNCTN_STAT | Functional Status at Registration |  |
| CAN_GENDER | Patient/s Gender | F, M |
| CAN_HGT_CM | Candidate/s Height - from WL for A & R Cands,TCR for L Cands |  |
| CAN_LISTING_DT | Listing Date - date/time candidate was physically added to the waiting list - this date/time does not change for transfers |  |
| CAN_MALIG | Any previous Malignancy | N, U, Y |
| CAN_MALIG_TY_BREAST | Previous Malignancy - Breast | 0.0, 1.0 |
| CAN_MALIG_TY_CNS_TUMOR | Previous Malignancy - CNS Tumor | 0.0, 1.0 |
| CAN_MALIG_TY_GENITOURINARY | Previous Malignancy - Genitourinary | 0.0, 1.0 |
| CAN_MALIG_TY_LEUK_LYMPH | Previous Malignancy - Leukemia/Lymphoma | 0.0, 1.0 |
| CAN_MALIG_TY_LIVER | Previous Malignancy - Liver | 0.0, 1.0 |
| CAN_MALIG_TY_LU | Previous Malignancy - Lung | 0.0, 1.0 |
| CAN_MALIG_TY_OTHER | Previous Malignancy - Other, specify | 0.0, 1.0 |
| CAN_MALIG_TY_SKIN_MEL | Previous Malignancy - Skin Melanoma | 0.0, 1.0 |
| CAN_MALIG_TY_SKIN_NON_MEL | Previous Malignancy - Skin Non-Melanoma | 0.0, 1.0 |
| CAN_MALIG_TY_THROAT | Previous Malignancy - Tongue/Throat/Larynx | 0.0, 1.0 |
| CAN_MALIG_TY_THYROID | Previous Malignancy - Thyroid | 0.0, 1.0 |
| CAN_MALIG_TY_UNK | Previous Malignancy - Type Unknown | 0.0, 1.0 |
| CAN_PERIPH_VASC | Symptomatic Peripheral Vascular Disease | N, U, Y |
| CAN_PREV_HL | Prev Heart-Lung Tx | 0.0, 1.0 |
| CAN_PREV_HR | Prev Heart Tx | 0.0, 1.0 |
| CAN_PREV_IN | Prev Intestine Tx | 0.0, 1.0 |
| CAN_PREV_KI | Prev Kidney Tx | 0.0, 1.0 |
| CAN_PREV_KI_TX_FUNCTN | If Candidate has had a prior Kidney Transplant, is the prior Kidney graft still functioning? | A, N, Y |
| CAN_PREV_KP | Prev Kidney-Pancreas Tx | 0.0, 1.0 |
| CAN_PREV_LI | Prev Liver Tx | 0.0, 1.0 |
| CAN_PREV_LU | Prev Lung Tx | 0.0, 1.0 |
| CAN_PREV_PA | Prev Pancreas Tx | 0.0, 1.0 |
| CAN_PREV_TX | Previous Transplants | 0.0, 1.0 |
| CAN_PRIMARY_PAY | Source of Payment//Primary | NAN, OTHER, OTHER PUBLIC INSURANCE, PRIVATE INSURANCE, PUBLIC INSURANCE - MEDICAID, PUBLIC INSURANCE - MEDICARE |
| CAN_TOT_ALBUMIN | Total Serum Albumin |  |
| CAN_WGT_KG | Candidate/s Weight - from WL for A & R Cands,TCR for L Cands |  |
| CAN_WORK_INCOME | Working for income | N, U, Y |
| LISTING_QUARTER | Quarter of the year for the date registered on waitlist | 1, 2, 3, 4 |
| OPTN_REGION | OPTN Region for Candidate's Permanent State | OTHER, REGION 1, REGION 10, REGION 11, REGION 2, REGION 3, REGION 4, REGION 5, REGION 6, REGION 7, REGION 8, REGION 9 |
| REC_FUNCTN_STAT | Pre-transplant Functional Status | None |

**Table S1.** Description of the variables considered for the predictive models. The categories of a categorical variable are shown in the categories column. If a variable is a numerical variable, its categories column will be empty.

| Feature | Percent Missing | Mean/Category Distribution |
| --- | --- | --- |
| AGE_AT_LISTING | 0% | 51.35 |
| CAN_ABO | 0% | A: 35.63%, A1: 1.08%, A1B: 0.13%, A2: 0.16%, A2B: 0.03%, AB: 5.56%, B: 13.21%, O: 44.21%, Reference Category: A |
| CAN_BMI | <0.01% | 28.47 |
| CAN_CITIZENSHIP | <0.01% | NON-RESIDENT ALIEN, YEAR ENTERED US: 0.3%, NON-US CITIZEN/NON-US RESIDENT, TRAVELED TO US FOR REASON OTHER THAN TRANSPLANT: 0.65%, NON-US CITIZEN/NON-US RESIDENT, TRAVELED TO US FOR TRANSPLANT: 0.09%, NON-US CITIZEN/US RESIDENT: 4.19%, RESIDENT ALIEN: 2.25%, US CITIZEN: 92.53%, NAN: <0.01%, Reference Category: NON_TO_RESIDENT_ALIEN_YEAR_ENTERED_US |
| CAN_DGN | 0.35% | GROUP_0: 3.05%, GROUP_1: 37.35%, GROUP_2: 39.99%, GROUP_3: 18.13%, GROUP_4: 1.49%, Reference Category: GROUP_0 |
| CAN_DIAB_TY | <0.01% | DIABETES STATUS UNKNOWN: 0.28%, NO: 62.83%, TYPE I: 3.39%, TYPE II: 32.04%, TYPE OTHER: 0.71%, TYPE UNKNOWN: 0.74%, NAN: <0.01%, Reference Category: DIABETES_STATUS_UNKNOWN |
| CAN_EDUCATION | <0.01% | ASSOCIATE/BACHELOR DEGREE: 16.59%, ATTENDED COLLEGE/TECHNICAL SCHOOL: 24.94%, GRADE SCHOOL (0-8): 6.34%, HIGH SCHOOL (9-12) OR GED: 41.11%, N/A (< 5 YRS OLD): 0.01%, NONE: 0.59%, POST-COLLEGE GRADUATE DEGREE: 6.53%, UNKNOWN: 3.88%, NAN: <0.01%, Reference Category: ASSOCIATE_BACHELOR_DEGREE |
| CAN_ETHNICITY_SRTR | 0% | LATINO: 17.02%, NON-LATINO OR UNKNOWN: 82.98%, Reference Category: LATINO |
| CAN_EXHAUST_PERIT_ACCESS | 0% | N: 95.71%, U: 3.98%, Y: 0.31%, Reference Category: N |
| CAN_EXHAUST_VASC_ACCESS | 0% | N: 95.9%, U: 3.79%, Y: 0.31%, Reference Category: N |
| CAN_FUNCTN_STAT | 3.02% | 78.04 |
| CAN_GENDER | 0% | F: 39.56%, M: 60.44%, Reference Category: F |
| CAN_HGT_CM | 0.01% | 170.03 |
| CAN_LISTING_DT | 0% | min date: 2007-01-02, max date: 2018-12-31 |
| CAN_MALIG | 0% | N: 91.73%, U: 0.71%, Y: 7.56%, Reference Category: N |
| CAN_MALIG_TY_BREAST | 0% | 0.0: 91.09%, 1.0: 8.91%, Reference Category: 0 |
| CAN_MALIG_TY_CNS_TUMOR | 0% | 0.0: 99.7%, 1.0: 0.3%, Reference Category: 0 |
| CAN_MALIG_TY_GENITOURINARY | 0% | 0.0: 80.22%, 1.0: 19.78%, Reference Category: 0 |
| CAN_MALIG_TY_LEUK_LYMPH | 0% | 0.0: 95.25%, 1.0: 4.75%, Reference Category: 0 |
| CAN_MALIG_TY_LIVER | 0% | 0.0: 96.84%, 1.0: 3.16%, Reference Category: 0 |
| CAN_MALIG_TY_LU | 0% | 0.0: 99.16%, 1.0: 0.84%, Reference Category: 0 |
| CAN_MALIG_TY_OTHER | 0% | 0.0: 63.44%, 1.0: 36.56%, Reference Category: 0 |
| CAN_MALIG_TY_SKIN_MEL | 0% | 0.0: 93.77%, 1.0: 6.23%, Reference Category: 0 |
| CAN_MALIG_TY_SKIN_NON_MEL | 0% | 0.0: 77.67%, 1.0: 22.33%, Reference Category: 0 |
| CAN_MALIG_TY_THROAT | 0% | 0.0: 99.26%, 1.0: 0.74%, Reference Category: 0 |
| CAN_MALIG_TY_THYROID | 0% | 0.0: 96.35%, 1.0: 3.65%, Reference Category: 0 |
| CAN_MALIG_TY_UNK | 0% | 0.0: 99.49%, 1.0: 0.51%, Reference Category: 0 |
| CAN_PERIPH_VASC | 0% | N: 90.67%, U: 0.98%, Y: 8.35%, Reference Category: N |
| CAN_PREV_HL | 0% | 0.0: 99.99%, 1.0: 0.01%, Reference Category: 0 |
| CAN_PREV_HR | 0% | 0.0: 99.55%, 1.0: 0.45%, Reference Category: 0 |
| CAN_PREV_IN | 0% | 0.0: 99.98%, 1.0: 0.02%, Reference Category: 0 |
| CAN_PREV_KI | 0% | 0.0: 87.69%, 1.0: 12.31%, Reference Category: 0 |
| CAN_PREV_KI_TX_FUNCTN | 0% | A: 0.1%, N: 0.73%, Y: 99.17%, Reference Category: A |
| CAN_PREV_KP | 0% | 0.0: 99.54%, 1.0: 0.46%, Reference Category: 0 |
| CAN_PREV_LI | 0% | 0.0: 98.49%, 1.0: 1.51%, Reference Category: 0 |
| CAN_PREV_LU | 0% | 0.0: 99.89%, 1.0: 0.11%, Reference Category: 0 |
| CAN_PREV_PA | 0% | 0.0: 99.43%, 1.0: 0.57%, Reference Category: 0 |
| CAN_PREV_TX | 0% | 0.0: 85.79%, 1.0: 14.21%, Reference Category: 0 |
| CAN_PRIMARY_PAY | <0.01% | OTHER: 0.33%, OTHER PUBLIC INSURANCE: 2.18%, PRIVATE INSURANCE: 38.86%, PUBLIC INSURANCE - MEDICAID: 7.58%, PUBLIC INSURANCE - MEDICARE: 51.05%, NAN: <0.01%, Reference Category: OTHER |
| CAN_TOT_ALBUMIN | 0% | 3.91 |
| CAN_WGT_KG | 0% | 82.74 |
| CAN_WORK_INCOME | 0% | N: 66.75%, U: 3.32%, Y: 29.93%, Reference Category: N |
| LISTING_QUARTER | 0% | 1: 24.85%, 2: 25.75%, 3: 25.1%, 4: 24.3%, Reference Category: 1 |
| OPTN_REGION | 0% | REGION 1: 3.53%, REGION 10: 8.47%, REGION 11: 11.27%, REGION 2: 11.46%, REGION 3: 15.24%, REGION 4: 9.36%, REGION 5: 15.91%, REGION 6: 3.9%, REGION 7: 7.54%, REGION 8: 6.18%, REGION 9: 6.84%, OTHER: 0.29%, Reference Category: REGION_1 |
| REC_FUNCTN_STAT | 0% | 75.54 |

**Table S2.** Summary statistics of the variables used in the analysis. The mean is shown for numerical variables and the category distribution is shown for categorical variables. We also show the percent of each variable missing, and the reference category that was dropped after one-hot-encoding.

| Category | Count |
| --- | --- |
| 80% - Normal activity with effort: some symptoms of disease | 34555 |
| 70% - Cares for self: unable to carry on normal activity or active work | 29711 |
| 90% - Able to carry on normal activity: minor symptoms of disease | 22063 |
| 60% - Requires occasional assistance but is able to care for needs | 11138 |
| 100% - Normal, no complaints, no evidence of disease | 9996 |
| 50% - Requires considerable assistance and frequent medical care | 5436 |
| 40% - Disabled: requires special care and assistance | 3020 |
| 20% - Very sick, hospitalization necessary: active treatment necessary | 1338 |
| 30% - Severely disabled: hospitalization is indicated, death not imminent | 790 |
| 10% - Moribund, fatal processes progressing rapidly | 354 |

**Table S3.** Pre-transplant functional status categories and counts.

| Category Name | New Grouping | Coefficient in Linear Regression |
| --- | --- | --- |
| HEPATORENAL SYNDROME | Group 0 | -14.4 |
| ACUTE TUBULAR NECROSIS | Group 0 | -9.41 |
| PRE-BMTRANSPLANTATION TOTAL BODY IRRADIATION | Group 0 | -5.77 |
| PA:DIABETES MELLITUS - TYPE I | Group 0 | -5.3 |
| GOUT | Group 0 | -5.0 |
| CANCER CHEMOTHERAPY INDUCED NEPHRITIS | Group 0 | -4.65 |
| CALCINEURIN INHIBITOR NEPHROTOXICITY | Group 0 | -4.27 |
| RENAL ARTERY THROMBOSIS | Group 0 | -4.26 |
| FABRY'S DISEASE | Group 0 | -4.1 |
| DIABETES - TYPE I NON-INSULIN DEP/JUV ON | Group 0 | -3.9 |
| LITHIUM TOXICITY | Group 0 | -3.88 |
| WILMS' TUMOR | Group 0 | -3.67 |
| DIABETES - TYPE I INSULIN DEP/JUV ONSET | Group 0 | -3.31 |
| DIABETES - TYPE II INSULIN DEP/ADULT ONS | Group 0 | -3.18 |
| DIABETES - TYPE II NON-INSULIN DEP/ADULT | Group 0 | -3.11 |
| POLYARTERITIS | Group 1 | -2.49 |
| DRUG RELATED INTERSTITIAL NEPHRITIS | Group 1 | -2.32 |
| SICKLE CELL ANEMIA | Group 1 | -2.16 |
| HIV NEPHROPATHY | Group 1 | -2.1 |
| SCLERODERMA | Group 1 | -1.9 |
| DIABETES MELLITUS - TYPE II | Group 1 | -1.9 |
| AMYLOIDOSIS | Group 1 | -1.89 |
| OTHER SPECIFY | Group 1 | -1.75 |
| ANALGESIC NEPHROPATHY | Group 1 | -1.74 |
| SARCOIDOSIS | Group 1 | -1.73 |
| nan | Group 1 | -1.6 |
| IDIO/POST-INF CRESCENTIC GLOMERULONEPHRI | Group 1 | -1.52 |
| OXALATE NEPHROPATHY (INCLUDES HEREDITARY OXALOSIS) | Group 1 | -1.46 |
| CHRONIC NEPHROSCLEROSIS-UNSPECIFIED | Group 1 | -1.45 |
| RENAL CELL CARCINOMA | Group 1 | -1.39 |
| HYPERTENSIVE NEPHROSCLEROSIS | Group 2 | -1.22 |
| DIABETES MELLITUS - TYPE I | Group 2 | -1.21 |
| HYPOPLASIA/DYSPLASIA/DYSGENSIS/AGENESIS | Group 2 | -1.15 |
| HEMOLYTIC UREMIC SYNDROME | Group 2 | -1.08 |
| MESANGIO-CAPILLARY 2 GLOMERULONEPHRITIS | Group 2 | -0.94 |
| MEDULLARY CYSTIC DISEASE | Group 2 | -0.92 |
| WEGENERS GRANULOMATOSIS | Group 2 | -0.79 |
| CHRONIC GLOMERULOSCLEROSIS UNSPECIFIED | Group 2 | -0.77 |
| CORTICAL NECROSIS | Group 2 | -0.77 |
| NEPHROLITHIASIS | Group 2 | -0.77 |
| PRUNE BELLY SYNDROME | Group 2 | -0.64 |
| MEMBRANOUS GLOMERULONEPHRITIS | Group 2 | -0.61 |
| SYSTEMIC LUPUS ERYTHEMATOSUS | Group 2 | -0.56 |
| RADIATION NEPHRITIS | Group 2 | -0.52 |
| POLYCYSTIC KIDNEYS | Group 2 | -0.51 |
| CONGENITAL OBSTRUCTIVE UROPATHY | Group 3 | -0.37 |
| CHRONIC GLOMERULONEPHRITIS UNSPECIFIED | Group 3 | -0.35 |
| NEPHRITIS | Group 3 | -0.3 |
| ANTIBIOTIC-INDUCED NEPHRITIS | Group 3 | -0.23 |
| RHEUMATOID ARTHRITIS | Group 3 | -0.15 |
| DIABETES MELLITUS - TYPE OTHER / UNKNOWN | Group 3 | -0.13 |
| IGA NEPHROPATHY | Group 3 | -0.13 |
| CHRONIC PYELONEPHRITIS/REFLUX NEPHROPATH | Group 3 | -0.12 |
| MYELOMA | Group 3 | -0.1 |
| FOCAL GLOMERULAR SCLEROSIS (FOCAL SEGMENTAL - FSG) | Group 3 | -0.1 |
| ACQUIRED OBSTRUCTIVE NEPHROPATHY | Group 3 | 0.0 |
| MESANGIO-CAPILLARY 1 GLOMERULONEPHRITIS | Group 3 | 0.03 |
| NEPHRONOPHTHISIS | Group 3 | 0.17 |
| MEMBRANOUS NEPHROPATHY | Group 3 | 0.26 |
| MALIGNANT HYPERTENSION | Group 3 | 0.41 |
| ALPORT'S SYNDROME | Group 4 | 0.58 |
| GOODPASTURE'S SYNDROME | Group 4 | 0.87 |
| FAMILIAL NEPHROPATHY | Group 4 | 0.99 |
| HENOCH-SCHOENLEIN PURPURA | Group 4 | 1.03 |
| RAPID PROGRESSIVE GLOMERULONEPHRITIS (RPGN) | Group 4 | 1.19 |
| UROLITHIASIS | Group 4 | 2.11 |
| CYSTINOSIS | Group 4 | 2.14 |
| THIN BASEMENT MEMBRANE DISEASE | Group 4 | 2.2 |
| ANTI-GBM | Group 4 | 2.31 |
| HEROIN NEPHROTOXICITY | Group 4 | 3.6 |
| CHOLESTEROL EMBOLIZATION | Group 4 | 3.84 |
| DYSPLASIA | Group 4 | 3.91 |
| PROGRESSIVE SYSTEMIC SCLEROSIS | Group 4 | 5.73 |
| INCIDENTAL CARCINOMA | Group 4 | 7.97 |
| LYMPHOMA | Group 4 | 8.06 |

**Table S4.** Original categories of primary diagnosis and their new grouping. We grouped the different categories together by first building a linear regression to predict pre-transplant functional status using functional status at registration and primary diagnosis. In the regression, primary diagnosis was encoded into binary variables. The regression then produced a coefficient for each of the different binary categories of primary diagnosis. We grouped these coefficients into five different groups using five equal-sized buckets. These five groups, corresponding to different primary diagnosis categories, become the new variable values. These groups represent different categories of primary diagnosis that have a similar pre-transplant functional status, controlling for functional status at registration. The regression was performed on the non out-of-time data and missing values were imputed using their median value. To avoid perfect multi collinearity, a primary diagnosis category was dropped prior to performing the linear regression. We assigned the dropped coefficient a value of 0 so it can be assigned to one of the five groups.

| Variable Name | Original Value | New Value |
| --- | --- | --- |
| CAN_PRIMARY_PAY | - ﻿Public insurance - Medicare & Choice - ﻿Public insurance - Medicare FFS (Fee for Service) - ﻿Public insurance - Medicare Unspecified | ﻿Public insurance - Medicare |
|  | - ﻿Public insurance - CHIP (Children's Health Insurance Program)" - ﻿Public insurance - Department of VA - ﻿Public insurance - Other government' | ﻿Other Public insurance |
|  | - ﻿Self - ﻿Pending - Free Care - ﻿US/State Govt Agency - ﻿Donation - ﻿Foreign Government Specify' | ﻿Other |

**Table S5.** Additional variable value groupings. These are values that have been grouped from their original value in the data into new values.

| Computation | Function | Package |
| --- | --- | --- |
| Elastic Net | ElasticNetCV | scikit-learn^44^ |
| Random Forests | RandomForestRegressor | scikit-learn |
| Support Vector Regression | SGDRegressor | scikit-learn |
| Gradient Boosting | ﻿GradientBoostingRegressor | scikit-learn |
| LightGBM | LGBMRegressor | lightgbm^30^ |
| XGBoost regression | XGBRegressor | xgboost^24^ |
| XGBoost classification | XGBClassifier | xgboost |
| GAM | LinearGAM | pygam^32^ |
| Ordinal Logistic Regression | LogisticAT | mord^45^ |
| Stacking Model | ﻿StackingCVRegressor | ﻿mlxtend^46^ |
| Friedman and Popescu’s *H* statistic | ﻿h_all_pairs | Sklearn-gbmi^47^ |
| Random Forest based imputation | ﻿MissForest | missingpy^48^ |
| Pruned Exact Linear Time | ﻿Pelt | ﻿ruptures^17^ |

**Table S6.** Functions and packages used for the major computations involved in the analysis.

| Validation | Final Model RMSE | Benchmark RMSE | Train Start (inclusive) | Train End (inclusive)/Validation Start (exclusive) | Test End (inclusive) | Train Size | Test Size |
| --- | --- | --- | --- | --- | --- | --- | --- |
| 1 | 12.8 | 14.38 | 2007-01-02 | 2007-08-11 | 2008-03-06 | 6937 | 6141 |
| 2 | 12.39 | 14.15 | 2007-01-02 | 2008-03-06 | 2008-09-30 | 13078 | 6225 |
| 3 | 12.16 | 13.72 | 2007-01-02 | 2008-09-30 | 2009-04-26 | 19303 | 5970 |
| 4 | 12.31 | 13.9 | 2007-01-02 | 2009-04-26 | 2009-11-20 | 25273 | 6385 |
| 5 | 12.35 | 13.93 | 2007-01-02 | 2009-11-20 | 2010-06-16 | 31658 | 6032 |
| 6 | 12.24 | 13.58 | 2007-01-02 | 2010-06-16 | 2011-01-10 | 37690 | 5898 |
| 7 | 12.29 | 13.6 | 2007-01-02 | 2011-01-10 | 2011-08-06 | 43588 | 5958 |
| 8 | 12.74 | 14.08 | 2007-01-02 | 2011-08-06 | 2012-03-01 | 49546 | 5844 |
| 9 | 12.84 | 14.19 | 2007-01-02 | 2012-03-01 | 2012-09-25 | 55390 | 5824 |
| 10 | 13.32 | 14.94 | 2007-01-02 | 2012-09-25 | 2013-04-21 | 61214 | 5768 |
| 11 | 13.53 | 15.23 | 2007-01-02 | 2013-04-21 | 2013-11-15 | 66982 | 6162 |
| 12 | 13.61 | 15.24 | 2007-01-02 | 2013-11-15 | 2014-06-11 | 73144 | 5695 |
| 13 | 13.78 | 15.19 | 2007-01-02 | 2014-06-11 | 2015-01-05 | 78839 | 5488 |
| 14 | 13.99 | 15.86 | 2007-01-02 | 2015-01-05 | 2015-08-01 | 84327 | 5518 |
| 15 | 13.38 | 15.18 | 2007-01-02 | 2015-08-01 | 2016-02-25 | 89845 | 5121 |
| 16 | 13.02 | 14.34 | 2007-01-02 | 2016-02-25 | 2016-09-20 | 94966 | 5362 |
| 17 | 12.93 | 14.28 | 2007-01-02 | 2016-09-20 | 2017-04-16 | 100328 | 5056 |
| 18 | 12.67 | 13.97 | 2007-01-02 | 2017-04-16 | 2017-11-10 | 105384 | 4602 |
| 19 | 12.53 | 13.68 | 2007-01-02 | 2017-11-10 | 2018-06-06 | 109986 | 4422 |
| 20 | 12.9 | 14.14 | 2007-01-02 | 2018-06-06 | 2018-12-31 | 114408 | 3993 |

**Table S7.** Additional cross validations of the final model compared to the benchmark model using 20 ROCV tests.

| RMSE Validation | σ RMSE Validation | Accuracy Within One Increment Validation | Accuracy Within Two Increments Validation | RMSE Train | Model Parameters |
| --- | --- | --- | --- | --- | --- |
| 12.99 | 0.682 | 80.19% | 94.24% | 11.81 | model:XGBoost, features:VIXGB Top 15, tree_method:auto, objective:reg:squarederror, booster:gbtree, learning_rate:0.01, gamma:0, max_depth:8, colsample_bytree:0.8, colsample_bynode:0.3, subsample:1, min_child_weight:1.0, n_estimators:500 |
| 13.02 | 0.709 | 80.51% | 94.18% | 12.18 | model:LightGBM, features:VIXGB Better than Random Variables, objective:regression, boosting_type:gbdt, learning_rate:0.1, num_leaves:32, min_child_samples:20, max_depth:-1, colsample_bytree:0.9, subsample:1, n_estimators:50 |
| 13.11 | 0.686 | 80.05% | 94.07% | 12.74 | model:GAM, features:VIXGB Top 10, max_iter:200, lam:0.9, n_splines:10 |
| 13.19 | 0.648 | 79.78% | 93.83% | 12.83 | model:elastic net, features: VIXGB Top 10, n_alphas:100, l1_ratio:1.0 |
| 13.28 | 0.698 | 80.14% | 93.92% | 12.85 | model:ordinal logistic regression, features:VIXGB Top 10, alpha:0.75 |
| 13.66 | 0.637 | 77.77% | 93.56% | 13.21 | model:random Forests, features:VIXGB Top 5, max_depth:12, min_samples_leaf:300, min_samples_split:947, n_estimators:500, max_features:sqrt, bootstrap:True |
| 13.91 | 0.954 | 78.95% | 92.11% | 13.36 | model:support vector regression, features:VIXGB Top 5, standardize:Nystroem, loss:epsilon_insensitive, penalty:l2, gamma:0.01, kernel:rbf, n_components:300, |
| 14.04 | 1.051 | 78.28% | 92.12% | 13.37 | model:XGBClassifier, features:VIXGB Top 5, tree_method:auto, objective:multi:softmax, booster:gbtree, learning_rate:0.01, gamma:0, max_depth:4, colsample_bytree:0.8, colsample_bynode:0.3, subsample:1, min_child_weight:0.4, n_estimators:500 |
| 14.5 | 0.784 | 77.65% | 91.05% | 14.22 | model:same as registration |
| 14.51 | 0.767 | 77.47% | 90.99% | 14.19 | model:average shift |

**Table S8.** Performance of the model with the top parameters from each model type using five ROCV tests.

| Segment | Size | RMSE | Accuracy Within One Increments | Accuracy Within Two Increments | Mean Pre-Transplant Functional Status | RMSE Benchmark Model |
| --- | --- | --- | --- | --- | --- | --- |
| AGE_AT_LISTING: $\leq$47.0 | 7936 | 12.22 | 82.69% | 95.22% | 73.67 | 13.5 |
| AGE_AT_LISTING: (47.0 - 60.0] | 8064 | 13.22 | 80.65% | 93.3% | 70.12 | 14.34 |
| AGE_AT_LISTING: $>$60.0 | 7667 | 13.37 | 81.14% | 93.34% | 69.81 | 14.48 |
| CAN_ABO: A | 8543 | 12.78 | 81.7% | 94.33% | 71.19 | 13.87 |
| CAN_ABO: A1 | 261 | 9.41 | 90.8% | 98.08% | 73.87 | 10.7 |
| CAN_ABO: A1B | 31 | 9.33 | 93.55% | 96.77% | 73.23 | 10.63 |
| CAN_ABO: A2 | 29 | 8.3 | 93.1% | 100.0% | 74.83 | 9.1 |
| CAN_ABO: AB | 1522 | 12.67 | 81.93% | 93.76% | 72.37 | 13.95 |
| CAN_ABO: B | 3210 | 13.15 | 82.24% | 93.24% | 70.99 | 14.72 |
| CAN_ABO: O | 10095 | 13.13 | 80.68% | 93.81% | 71.07 | 14.21 |
| CAN_BMI: $\leq$26.0 | 9200 | 12.98 | 81.25% | 93.8% | 71.53 | 14.24 |
| CAN_BMI: (26.0 - 31.0] | 7452 | 13.09 | 81.33% | 94.06% | 71.47 | 14.26 |
| CAN_BMI: $>$31.0 | 7046 | 12.73 | 81.96% | 94.05% | 70.58 | 13.77 |
| CAN_DGN: GROUP_0 | 1313 | 19.91 | 58.42% | 79.74% | 51.44 | 21.33 |
| CAN_DGN: GROUP_1 | 9404 | 13.13 | 81.66% | 93.77% | 70.46 | 14.38 |
| CAN_DGN: GROUP_2 | 8725 | 11.79 | 83.47% | 95.58% | 73.2 | 12.94 |
| CAN_DGN: GROUP_3 | 3912 | 11.98 | 84.28% | 95.5% | 74.92 | 12.98 |
| CAN_DGN: GROUP_4 | 347 | 11.51 | 83.86% | 96.54% | 75.71 | 11.87 |
| CAN_EDUCATION: ASSOCIATE/BACHELOR DEGREE | 4425 | 13.06 | 81.58% | 94.08% | 72.4 | 14.47 |
| CAN_EDUCATION: ATTENDED COLLEGE/TECHNICAL SCHOOL | 6109 | 12.82 | 81.86% | 93.96% | 71.52 | 13.83 |
| CAN_EDUCATION: GRADE SCHOOL (0-8) | 1451 | 13.29 | 80.08% | 94.21% | 70.17 | 14.19 |
| CAN_EDUCATION: HIGH SCHOOL (9-12) OR GED | 9285 | 12.79 | 81.49% | 94.24% | 70.7 | 13.83 |
| CAN_EDUCATION: NONE | 129 | 13.21 | 80.62% | 92.25% | 68.22 | 15.7 |
| CAN_EDUCATION: POST-COLLEGE GRADUATE DEGREE | 1734 | 12.53 | 84.6% | 93.89% | 72.96 | 13.87 |
| CAN_EDUCATION: UNKNOWN | 566 | 15.79 | 71.55% | 88.52% | 65.58 | 18.29 |
| CAN_ETHNICITY_SRTR: LATINO | 4415 | 13.23 | 80.18% | 93.98% | 72.72 | 14.21 |
| CAN_ETHNICITY_SRTR: NON-LATINO OR UNKNOWN | 19286 | 12.88 | 81.78% | 93.96% | 70.89 | 14.09 |
| CAN_FUNCTN_STAT: $\leq$70 | 11770 | 13.4 | 79.11% | 93.33% | 63.5 | 14.07 |
| CAN_FUNCTN_STAT: (70 - 80] | 6119 | 11.39 | 86.48% | 95.21% | 76.18 | 11.6 |
| CAN_FUNCTN_STAT: $>$80 | 5509 | 13.23 | 81.74% | 94.48% | 82.61 | 16.26 |
| CAN_HGT_CM: $\leq$165.1 | 8937 | 12.87 | 81.95% | 94.08% | 71.82 | 14.08 |
| CAN_HGT_CM: (165.1 - 175.26] | 7826 | 12.83 | 81.83% | 94.25% | 71.29 | 13.91 |
| CAN_HGT_CM: $>$175.26 | 6928 | 13.15 | 80.51% | 93.48% | 70.4 | 14.37 |
| CAN_PERIPH_VASC: N | 20843 | 12.98 | 81.38% | 93.94% | 71.38 | 14.17 |
| CAN_PERIPH_VASC: U | 195 | 14.0 | 77.44% | 93.33% | 76.92 | 15.24 |
| CAN_PERIPH_VASC: Y | 2663 | 12.5 | 82.65% | 94.37% | 69.58 | 13.49 |
| CAN_PREV_LI: 0.0 | 23245 | 12.85 | 81.76% | 94.12% | 71.41 | 13.99 |
| CAN_PREV_LI: 1.0 | 456 | 16.95 | 67.54% | 85.53% | 61.91 | 19.28 |
| CAN_PRIMARY_PAY: OTHER | 44 | 16.03 | 72.73% | 86.36% | 62.27 | 16.85 |
| CAN_PRIMARY_PAY: OTHER PUBLIC INSURANCE | 552 | 12.43 | 83.33% | 95.29% | 74.22 | 13.54 |
| CAN_PRIMARY_PAY: PRIVATE INSURANCE | 7595 | 13.74 | 79.96% | 92.81% | 72.11 | 15.32 |
| CAN_PRIMARY_PAY: PUBLIC INSURANCE - MEDICAID | 2067 | 13.32 | 79.49% | 93.71% | 69.23 | 14.18 |
| CAN_PRIMARY_PAY: PUBLIC INSURANCE - MEDICARE | 13441 | 12.41 | 82.63% | 94.64% | 70.94 | 13.38 |
| CAN_TOT_ALBUMIN: $\leq$3.8 | 9363 | 14.44 | 77.74% | 91.56% | 67.53 | 15.74 |
| CAN_TOT_ALBUMIN: (3.8 - 4.2] | 7125 | 12.27 | 82.96% | 94.98% | 73.02 | 13.41 |
| CAN_TOT_ALBUMIN: $>$4.2 | 7212 | 11.44 | 84.87% | 96.09% | 74.26 | 12.44 |
| CAN_WGT_KG: $\leq$73.0 | 7926 | 12.94 | 81.35% | 94.01% | 71.79 | 14.23 |
| CAN_WGT_KG: (73.0 - 90.26] | 7873 | 12.99 | 81.56% | 94.06% | 71.22 | 14.14 |
| CAN_WGT_KG: $>$90.26 | 7901 | 12.89 | 81.53% | 93.85% | 70.67 | 13.96 |
| CAN_WORK_INCOME: N | 16386 | 13.17 | 80.62% | 93.56% | 69.19 | 14.32 |
| CAN_WORK_INCOME: U | 524 | 15.89 | 69.66% | 90.46% | 65.82 | 17.52 |
| CAN_WORK_INCOME: Y | 6791 | 12.09 | 84.49% | 95.26% | 76.56 | 13.27 |
| OPTN_REGION: OTHER | 52 | 13.59 | 82.69% | 90.38% | 67.12 | 13.3 |
| OPTN_REGION: REGION 1 | 711 | 12.51 | 82.42% | 94.23% | 70.75 | 12.66 |
| OPTN_REGION: REGION 10 | 2008 | 12.34 | 82.92% | 94.57% | 69.11 | 12.94 |
| OPTN_REGION: REGION 11 | 2599 | 11.27 | 86.26% | 96.23% | 70.98 | 12.61 |
| OPTN_REGION: REGION 2 | 2422 | 13.77 | 79.03% | 92.11% | 71.16 | 15.41 |
| OPTN_REGION: REGION 3 | 3869 | 11.6 | 84.23% | 95.58% | 68.07 | 12.6 |
| OPTN_REGION: REGION 4 | 2394 | 13.74 | 80.41% | 93.65% | 72.3 | 14.56 |
| OPTN_REGION: REGION 5 | 3699 | 15.01 | 75.16% | 91.29% | 75.36 | 17.05 |
| OPTN_REGION: REGION 6 | 964 | 12.89 | 81.85% | 94.19% | 74.3 | 14.85 |
| OPTN_REGION: REGION 7 | 1641 | 13.71 | 78.92% | 92.38% | 68.63 | 15.08 |
| OPTN_REGION: REGION 8 | 1602 | 13.39 | 78.84% | 93.51% | 68.71 | 13.9 |
| OPTN_REGION: REGION 9 | 1740 | 10.45 | 89.31% | 96.84% | 74.28 | 10.13 |

**Table S9.** Performance of the final model on different segments of the population in the out-of-time data. For each categorical variable, the performance was tested on each of the different variable categories and for numerical variables, the performance was tested on the first third, second third, on last third percentiles of values.
